# On Sulphite of Magnesia

**Published:** 1868

**Authors:** Joseph P. Remington

**Affiliations:** Brooklyn, N. Y.


### On Sulphite of Magnesia. By Joseph P. Remington, Brooklyn, N. Y.

This salt having been in request lately, the authorities at the writer's command were searched for a formula for its preparation. It did not appear to be a frequent subject for examination, and the published investigations seemed rather meagre and wanting in details.

The only formula appearing was one from Wittstein's Vierteljahresschrift, as follows: "Sulphite of Magnesia may be prepared by passing sulphurous acid gas through water holding carbonate of magnesia in suspension; but the salt so obtained is not quite white. A better way is to dissolve 136 parts of crystalized sulphite of soda, free from carbonate and sulphate, in the smallest quantity of hot water, and to filter into this hot liquid a concentrated solution of 123 parts of epsom salts, the mixture to be stirred until cold. The mass of fine crystals which form are allowed to drain on a strainer, then pressed and dried at a moderate heat. The product should weigh 69 parts."

It is not an uncommon occurrence to find the sulphite of soda of commerce contaminated with sulphate and carbonate; and, if made with anything but a freshly prepared sulphite of soda, the yield would be diminished in proportion to the extent of the contamination. There is besides, in the above process, a small yield of the sulphite of magnesia, compared with the quantities of the two salts taken, and the yield was much less than that stated in the writer's hands. Having had the same experience with regard to saturating the carbonate, suspended in water, with sulphurous acid gas, a somewhat different plan was adopted.

Five grammes Jenning's Magnesia (re-calcined) was made into a thick, *smooth* paste, with 10 c.c. of distilled water; to this was added slowly, with stirring, 102 c.c. of aqueous sulphurous acid, sp. gr. 1·037. The liquid on the surface now showed an acid reaction, and had a yellow color. After standing a few minutes, the supernatant liquid was decanted. The crystals were then placed on a tarred filter and washed with distilled water until the washings came through colorless; and a small portion, on being tested with chloride of barium, produced a precipitate which was almost entirely dissolved by hydrochloric acid. The filter and contents were then dried at a temperature not exceeding 100 deg. F., and weighed 15·310 grammes, which was 3·06 times the weight of the magnesia used, or 306·2 per cent.; the theoretical yield should have been 3·95 times the quantity of magnesia started with.* The loss is owing principally to over saturation, as sulphite of magnesia is very soluble in aqueous sulphurous acid; but it was thought to be best to secure the conversion of the whole of the magnesia by over saturating, since the excess of sulphurous acid can easily be disposed of by decantation, and after draining and washing, the crystals are, of course, free from acid. The sulphite of magnesia, as obtained, was in very small white crystals, having the peculiar taste of the sulphites, though, on account of its insolubility, the taste was not so disagreeable as that of the more soluble sulphites of soda and potassa. The process was tried on a somewhat larger scale, and succeeded. Eight ounces av. of Jenning's Calcined Magnesia was made into a smooth paste with a pint of distilled water and aqueous sulphurous acid U. S. P., sp. gr. 1·035 was added, with

*This is the average of several experiments, all conducted on the same plan and with the same quantities, and represents about what the results would be in ordinary practice.

stirring, until the liquid gave a slight acid reaction; the crystals formed were then allowed to subside, and the clear liquid was decanted; the sulphite of magnesia was then drained on a muslin strainer and washed with distilled water until free from impurities, then again allowed to drain, and dried on bibulous paper; the yield was 1 lb. 8 oz. of dry crystals. The washing can be accomplished most effectually, and with the use of the least water, by allowing the crystals to collect in a stratum on the bottom of the strainer, and then adding just enough distilled water to cover the surface; any sulphate of magnesia is dissolved, and this, together with the yellow mother water, is displaced by the descending clean water, and the salt is left perfectly white. By this process, sulphite of magnesia can be obtained as pure and white as by double decomposition, with economy in the most valuable items, time and labor, the yellow coloration all disappearing by the simple process of washing, and the loss in washing is small, as the salt is difficultly soluble in cold water. The yellow color seems to be caused by an impurity soluble in sulphurous acid (believed to be iron) and it only appears when the acid is in excess, and is, therefore, a good indication that the magnesia is all converted into sulphite. When made from Henry's Magnesia, no yellow color was visible during any step in the process. It is almost needless to say that it was not found profitable to evaporate and re-crystalize the mother water.

